# Curative Effect of AD-MSCs against Cisplatin-Induced Hepatotoxicity in Rats is Potentiated by Azilsartan: Targeting Oxidative Stress, MAPK, and Apoptosis Signaling Pathways

**DOI:** 10.1155/2023/6767735

**Published:** 2023-10-23

**Authors:** Amany Abdlrehim Bekhit, Olivia N. Beshay, Michael A. Fawzy, Sara Mohamed Naguib Abdel-Hafez, Gaber El-Saber Batiha, Farid S. Ataya, Moustafa Fathy

**Affiliations:** ^1^Department of Biochemistry, Faculty of Pharmacy, Minia University, Minia 61519, Egypt; ^2^Department of Histology and Cell Biology, Faculty of Medicine, Minia University, Minia 61519, Egypt; ^3^Department of Pharmacology and Therapeutics, Faculty of Veterinary Medicine, Damanhour University, Damanhour, AlBeheira 22511, Egypt; ^4^Department of Biochemistry, College of Science, King Saud University, P.O. Box, 2455, Riyadh 11451, Saudi Arabia; ^5^Department of Regenerative Medicine, Graduate School of Medicine and Pharmaceutical Sciences, University of Toyama, Toyama 930-0194, Japan

## Abstract

Despite its clinical value, cisplatin (CISP) is complicated by marked hepatotoxicity via inducing oxidative stress, inflammatory, and apoptotic pathways. This study aims to explore the protective impact of azilsartan (AZIL), an antihypertensive drug, in addition to adipose tissue-derived mesenchymal stem cells (AD-MSCs) on CISP-induced hepatotoxicity. After characterization and labeling of AD-MSCs by PKH26 dye, 54 Wistar male albino rats were randomly divided into nine groups: I (CONT), II (AZIL.H), III (CISP), IV (CISP + AZIL.L), V (CISP + AZIL.H), VI (CISP + AD-MSCs), VII (CISP + AZIL.L + AD-MSCs), VIII (CISP + AZIL.H + AD-MSCs), and IX (CISP + VITA C). Serum alanine aminotransferase (ALT), alanine aminotransferase (AST), and albumin levels were determined. Assessment of reactive oxygen species, malondialdehyde, and glutathione contents, and superoxide dismutase activity and histopathological evaluations were done on hepatic tissue. Quantitative real-time PCR was utilized to estimate the expression of *TNF-α* and *IL-6* genes. Cell homing of labeled AD-MSCs to the liver tissues was investigated. Hepatic expression of JNK1/2, ERK1/2, p38, Bax, Bcl-2, and cleaved caspase-3 proteins was investigated by western blot analysis. CISP elevated serum ALT and AST activities, reduced albumin level, and remarkably changed the hepatic architecture. It increased the expression *TNF-α* and *IL-6* genes, raised the expression of JNK1/2, ERK1/2, p38, Bax, and cleaved caspase-3 proteins, and diminished the Bcl-2 protein. By contrast, treatment of animals with either AZIL or AD-MSCs dramatically reduced the effects of CISP injection. Moreover, treatment with combination therapy (AZIL.L or H + AD-MSCs) considerably mitigated all previously mentioned alterations superior to AZIL or AD-MSCs alone, which might be attributed to the AZIL-enhanced homing ability of AD-MSCs into the injured liver tissue. In conclusion, the present findings demonstrated that AZIL improves the hepatoprotective potential of AD-MSCs against CISP-induced hepatotoxicity by modulating oxidative stress, mitogen-activated protein kinase, and apoptotic pathways.

## 1. Introduction

Cisplatin (CISP) is regarded as one of the most valuable anticancer drugs and has a remarkable impact in the treatment of a vast range of malignancies, such as ovarian, testicular, breast, colorectal, and lung cancers [1]. Unfortunately, the usefulness of CISP is hampered due to its common complications, including nephrotoxicity, hepatotoxicity, cardiotoxicity, and ototoxicity [2]. Liver toxicity has been documented as a deleterious effect of CISP use [3].

Traditionally, CISP treatment triggers reactive oxygen species (ROS), which could damage either tumor cells or other cells, such as liver cells, without distinction [4]. Overproduction of ROS, which causes the development of CISP-induced hepatotoxicity, is thought to be mediated by several processes, including oxidative stress, inflammation, and apoptosis [5]. Moreover, the representatives of the mitogen-activated protein kinases (MAPKs) family, namely extracellular signal-regulated kinase (ERK), c-Jun N-terminal kinase (JNK), and p38 have been implicated in mediating various injuries, including CISP-induced liver toxicity [6].

Cell-based therapies are emerging as a viable alternative to traditional pharmaceutical treatments [7–9]. Mesenchymal stem cells (MSCs) provide several advantages in regenerative medicine, like multidifferentiation potential, minimal immunogenicity, and a higher proliferation rate. Furthermore, they can migrate towards the microenvironments of damaged areas [10–12]. Regarding hepatic disorders, stem cell transplantation has recently been used as an additional therapeutic strategy. Numerous studies using adipose tissue-derived MSCs (AD-MSCs) have shown enhanced liver histopathology and function [13, 14]. In addition, the easy arrival to subcutaneous AD, its uncomplicated isolation procedures, and the reproducibility of sample collection render AD the most appealing source of MSCs [15].

However, maintaining their characters and selecting the optimum conditions for their therapeutic efficacy is somewhat challenging [16, 17]. As a result, recent investigations have focused on designing strategies for increasing MSC therapeutic efficacy through the use of either natural [18] or chemical substances [19] or by altering culture conditions [20].

Azilsartan (AZIL), a novel angiotensin receptor blocker, has the strongest antihypertensive activity when compared to other drugs in the same family [21]. Previous researches demonstrate other pharmacological actions and pleiotropic health benefits of AZIL on endothelial dysfunction, cerebral ischemia, breast cancer, renal ischemia, and lung injury [22–26]. Besides, it has been demonstrated that AZIL has the ability to protect the liver against nonalcoholic liver disease triggered by high-fat consumption [27]. Yet, it is not reported whether AZIL possesses a protective effect against CISP-induced hepatotoxicity or not, as well as its effect on the potential hepatoprotective potential of AD-MSCs.

Repurposing drugs and looking for new pharmacological actions for synthetic [28, 29] or natural [30] candidates have attracted great attention [31–33]. Regardless of the evidence that cytotherapy with AD-MSCs leads to protection against CISP-evoked nephrotoxicity [34–36], the effect of AD-MSCs on CISP-evoked hepatotoxicity seems questionable, with a lack of investigations tackling this issue. Until now, only one study has demonstrated the hepatoprotective effect of AD-MSCs on CISP-evoked hepatotoxicity via modulating TGF-*β*1/Smad and PI3K/AKT signaling pathways and highlighted the importance of autophagy in potentiating this effect of stem cell-based therapy [37]. So, the objective of this study is to assess the hepatoprotective impact of AZIL and AD-MSCs against CISP-induced hepatotoxicity in rats when administered separately or simultaneously and demonstrate the possible underlying mechanism by investigating oxidative stress, MAPK, and apoptotic signaling pathways that may be implicated in this outcome.

## 2. Materials and Methods

### 2.1. Drugs and Chemicals

CISP was obtained from Mylan Institutional LLC- (Rockford, USA). AZIL powder (Rameda Pharmaceutical Co., Giza, Egypt) was freshly prepared in a 0.5% w/v carboxy methylcellulose solution. VITA C powder was obtained from (Adwia Pharmaceuticals, Cairo, Egypt). The rest of the chemicals utilized were of the highest analytical grades available.

### 2.2. Preparation of AD-MSCs

The inguinal subcutaneous adipose tissue of male Wistar rats was carefully dissected, excised, and divided into 0.5 mm^3^ fragments. Then, Hank's balanced salt solution with constant agitation for 1 hr at 37°C was utilized to enzymatically digest the homogenized adipose tissues utilizing 0.075% collagenase II (SERVA Electrophoresis GmbH, Heidelberg, Germany). Filtration, centrifugation, and erythrocyte lysis buffer were applied to the cell suspension. The cells were then extracted for cell culture in Dulbecco's modified Eagle's medium (DMEM) with 10% bovine serum (Gibco/BRL), 1.25 mg/L amphotericin B (Gibco/BRL), and 1% penicillin–streptomycin (Gibco/BRL). Cells that weren't adherent were eliminated by a PBS wash. In DMEM with 10% FBS, 1.25 mg/L of amphotericin B (Gibco/BRL), and 1% of penicillin–streptomycin (Gibco/BRL), adherent cells were resuspended. PBS was employed to wash the cells twice and treated with 0.25% trypsin in 1 mM EDTA (Gibco/BRL) at 37°C for 5 min once they had reached 80%–90% confluence. Cells were resuspended in serum-supplemented medium following centrifugation, and they were then placed in a 50 cm^2^ culture flask. Following the third passage, the obtained cultures were applied to transplantation as previously described [38].

### 2.3. Characterization of MSCs and Labeling with PKH26 Dye

Detection of the positive expression of MSCs biomarkers (CD73, CD105, and CD90) with negative expression of hematopoietic biomarkers (CD45 and CD34) was used to characterize AD-MSCs in culture utilizing a flow cytometer. In brief, after blocking with 5% bovine serum albumin in PBS for 30 min at room temperature, cells were incubated with fluorescein isothiocyanate or phycoerythrin-conjugated antibodies for an hour at room temperature [39]. Antibodies for CD90, CD105, CD34, and CD45 (Beckman Coulter, Brea, CA, USA) as well as CD73 (BD Pharmingen, Franklin Lakes, NJ, USA) were utilized.

To assess the hepatic homing of MSCs, they were labeled with PKH26 fluorescent dye (Sigma–Aldrich, Saint Louis, MO, USA) in compliance with the supplier's protocol. MSCs were administered to rats by intravenous route after being pelleted and suspended in a dye solution. At the final stage of the investigation, hepatic tissues were placed in 10% formalin. To confirm migration, liver sections were inspected under a fluorescence microscope [40].

The average count of PKH26-labeled AD-MSCs were counted per section in 10 randomly nonoverlapping microscopic fields using power × 200 magnifications of the sections from each rat [41]. The results were carried out utilizing the Image-J/NIH software.

### 2.4. Animals

This study was performed on male Wistar albino rats (*n* = 54, 170–200 g, 6–8 weeks old) that were taken from the National Research Center (Giza, Egypt). Animals were maintained in cages with unrestricted availability to food and water for 14 days before starting the study to allow them to acclimate to the laboratory conditions. Rats were cared for in accordance with the Declaration of Helsinki's guidelines, which were approved by the Research Ethics Committee of Minia University, Egypt (ES04/2021).

### 2.5. Study Design

**Figure 1:**
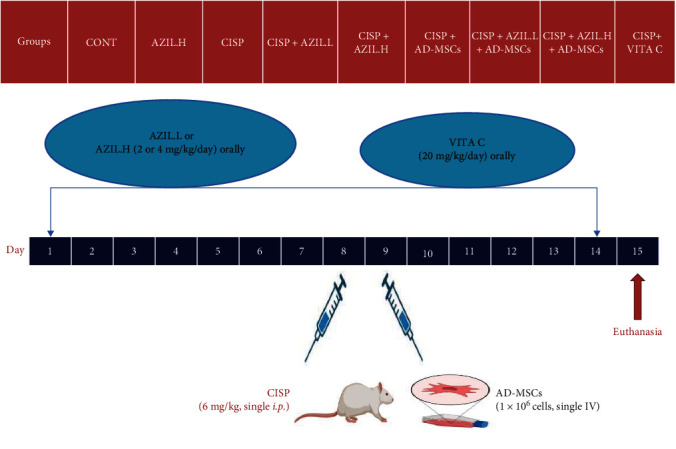
Schematic diagram of the study design.

Nine groups with six rats in each group were incorporated in this experiment and allocated randomly as follows (Figure 1):

  Group I (Control (CONT)): rats administered (0.5% carboxy methylcellulose) orally for 2 weeks plus a single intraperitoneal (*i.p*.) dose of 0.9% sodium chloride on the eighth day.
  Group II (AZIL.High (H)): rats orally administered AZIL (4 mg/kg) [23] once a day for 2 weeks.
  Group III (CISP): rats administered (0.5% carboxy methylcellulose) orally for 2 weeks plus a single *i.p*. dose of CISP (6 mg/kg) [42, 43] on the eighth day.
  Group IV (CISP + AZIL.Low (L)): rats administered AZIL.L (2 mg/kg/day) [23] orally for 2 weeks plus a single *i.p*. dose of CISP (6 mg/kg) on the eighth day.
  Group V (CISP + AZIL.H): rats administered AZIL.H (4 mg/kg/day) orally for 2 weeks plus a single *i.p*. dose of CISP (6 mg/kg) on the eighth day.
  Group VI (CISP + AD-MSCs): rats administered a single *i.p*. dose of CISP (6 mg/kg) plus AD-MSCs (1 × 10^6^ cells) [37] by intravenous injection 1 day following the CISP dose.
  Group VII (CISP + AZIL.L + AD-MSCs): rats administered AZIL.L (2 mg/kg/day) orally for 2 weeks plus a single *i.p*. dose of CISP (6 mg/kg) on the eighth day and AD-MSCs (1 × 10^6^ cells) by intravenous injection 1 day following the CISP dose.
  Group VIII (CISP + AZIL.H + AD-MSCs): rats administered AZIL.H (4 mg/kg/day) orally for 2 weeks plus a single *i.p*. dose of CISP (6 mg/kg) on the eighth day and AD-MSCs (1 × 10^6^ cells) by intravenous injection 1 day following the CISP dose.
  Group IX (CISP + Vitamin C (VITA C)) group: rats administered VITA C (20 mg/kg/day) [44] orally for 2 weeks plus a single *i.p*. dose of CISP (6 mg/kg) on the eighth day. CISP + VITA C was used as a positive CONT group.

### 2.6. Samples Collection

After 7 days of CISP administration, rats were weighed, anesthetized with isoflurane, and euthanized. Blood samples were obtained from each rat and then centrifuged for serum collection. Sera are utilized for the detection of alanine aminotransferase (ALT), alanine aminotransferase (AST), and albumin levels. Liver tissues were isolated, rinsed thoroughly with isotonic saline solution, dried utilizing filter paper, and weighed to calculate the liver index based on the following equation: (liver weight/body weight) × 100 [45]. Then, portions of liver specimens were homogenized in cold potassium phosphate buffer (0.05 M, pH 7.4) and then centrifuged to collect the supernatant in order to estimate oxidative stress markers. Other liver portions were fixed in 10% formalin for histological investigation, while the remaining portions were utilized for qRT-PCR and western blot analysis.

### 2.7. Assessment of Serum Biomarkers

Serum levels of ALT and AST were estimated utilizing kits supplied by BioMed Diagnostics (Badr City, Egypt) with catalog numbers (GPT113100 and GOT111060), respectively. Albumin level was assessed in the serum samples using an albumin kit (catalog number: 210 001), obtained from Spectrum Diagnostics (Cairo, Egypt). All procedures were carried out in accordance with the manufacturer's guidelines.

### 2.8. Assessment of Hepatic ROS Level and Oxidative Stress Biomarkers

ROS were detected by mixing the samples with the fluorescent probe H_2_DCF-DA (Sigma, St. Louis, MO, USA), followed by incubation at 37°C for 30 min, and measuring the fluorescence intensity at excitation 490 nm and emission 540 nm by using a microplate reader [46]. Malondialdehyde (MDA) content (MD 25 29), reduced glutathione (GSH) level (GR 25 11), and superoxide dismutase (SOD) activity (SD 25 21) in liver tissues were determined by specific kits procured from Biodiagnostics Co. (Giza, Egypt) following the supplier's guidance.

### 2.9. Gene Expression Assessment

Total RNA was extracted from frozen liver specimens applying SV Total RNA Isolation Kit (#Z3105, Promega, Madison, WI, USA) following the product's recommendations, and then cDNAs were synthesized utilizing a high-capacity cDNA reverse transcription kit (#4374966, Applied Biosystems, Thermo Fisher Scientific, Waltham, MA, USA) as recommended by the manufacturer. For quantitative PCR, assays were carried out utilizing SYBR Green qPCR Master Mix (2X) (#Ab179461, Thermo Scientific Fermentas, St. Leon-Ro, Germany) with appropriate primers in a StepOnePlus™ Real-Time PCR system (Applied Biosystems, Waltham, MA, USA). The specific sequence of primers utilized was as follows: tumor necrosis factor-alpha (*TNF-α*) forward primer, 5′-TGATCCGAGATGTGGAACTG-3′ and reverse primer, 5′-GGCCATGGAACTGATGAGAG-3′, interleukin (*IL*)-*6* forward primer, 5′-GCCCTTCAGGAACAGCTATGA-3′ and reverse primer, 5′-TGTCAACAACATCAGTCCCAAGA-3′, and *β-actin* forward primer, 5′-AGGCATCCTCACCCTGAAGTA-3′ and reverse primer, 5′-CACACG CAGCTCATTGTAGA-3′. The SYBR green results were normalized to *β*-actin as a housekeeping gene. Fold changes of *TNF-α* and *IL-6* genes expression were evaluated utilizing the *ΔΔ*CT formula [47] and displayed relative to the CONT samples.

### 2.10. Western Blot Assessment

For estimation of p-JNK1/2/total JNK1/2, p-ERK1/2/total ERK1/2, p-P38/total P38, Bax, Bcl-2, and cleaved caspase-3 expressions in liver tissue, Western blot analysis was conducted as described before [48]. In brief, equivalent amounts of protein extract (50 *µ*g) from all studied groups were loaded onto 12.5% SDS-polyacrylamide gels, and separated proteins were shifted to PVDF membranes. Incubation with 5% (w/v) nonfat dry milk for an hour at room temperature was performed to block the membranes, followed by incubation with primary antibodies against p-JNK (#44-682G, Thermo Fisher Scientific), JNK (#AHO1362, Thermo Fisher Scientific), p-ERK (#4370, Cell Signaling Technology), ERK (#9102, Cell Signaling Technology), p-p-38 (#4511, Cell Signaling Technology), p-38 (#8690, Cell Signaling Technology), Bcl-2-associated X protein (Bax) (#2772, Cell Signaling Technology), B-cell lymphoma 2 (Bcl-2) (#PA5-27094, Thermo Fisher Scientific), cleaved caspase-3 (#9661, Cell Signaling Technology), and *β*-actin (#4970, Cell Signaling Technology) at 4°C overnight. For membranes, a 30–60 min washing time was used, accompanied by incubation with the suitable secondary antibodies for one hour. Enhanced chemiluminescence substrates (Amersham Bioscience, Freiburg, Germany) were applied to blots to visualize bands. The Gel-Pro Analysis 7.0 tool (Media Cybernetics, Rockville, MD, USA) was employed to quantify the detected bands following normalization to *β*-actin and relative to the CONT samples.

### 2.11. Histological Assessment

Formalin-fixed liver specimens were dehydrated, cleared in xylene, and embedded in paraffin. Five micrometers thick liver slices were cut, stained with Hematoxylin and Eosin stain, and examined to study the pathological alterations [49] using an Olympus (U.TV0.5XC-3) light microscope.

### 2.12. Morphometric Analysis

Evaluations were performed utilizing Image-J/NIH software. Ten nonoverlapping microscopic fields using power × 400 magnifications of the sections from each rat [50] were inspected for detection of the following items:

1. The average count of inflammatory cell infiltration.
2. The average count of apoptotic cells.

### 2.13. Statistical Analysis

GraphPad prism® version 6 (GraphPad Software Inc., La Jolla, CA, USA) was employed for data analysis. The results of the current study were shown as the mean ± SEM. A one-way ANOVA with a post hoc Tukey's test was utilized to recognize the significance of differences among the studied groups. The levels of significance were recorded when probability values were less than 0.05.

## 3. Results

### 3.1. Characterization of Cell Surface Markers Expression in the Isolated AD-MSCs

**Figure 2:**
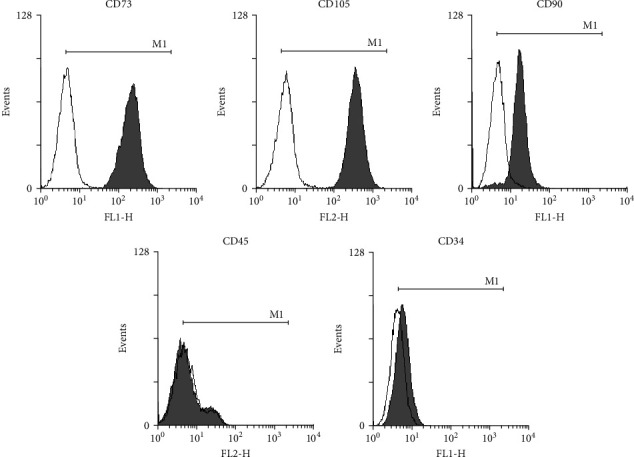
The characterization of cell surface markers expression in the isolated AD-MSCs was evaluated by flow cytometry. AD-MSCs were markedly positive for MSCs special biomarkers, including CD73, CD105, and CD90. While being negative for the hematopoietic biomarkers CD45 and CD34.

Flow cytometric analysis was employed to identify the cultured cells at passage three. As seen in Figure 2, 96.6% ± 1.2%, 95.33% ± 1.8%, and 88.0% ± 2.0% of the cultured AD-MSCs expressed CD73, CD105, and CD90, respectively. Contrarily, just 1.8% ± 0.1% and 3.3% ± 0.8% of them expressed the hematopoietic biomarkers CD45 and CD34, respectively.

### 3.2. Effects of AZIL and AD-MSCs on Liver Index and Serum Levels of ALT, AST, and Albumin

**Figure 3:**
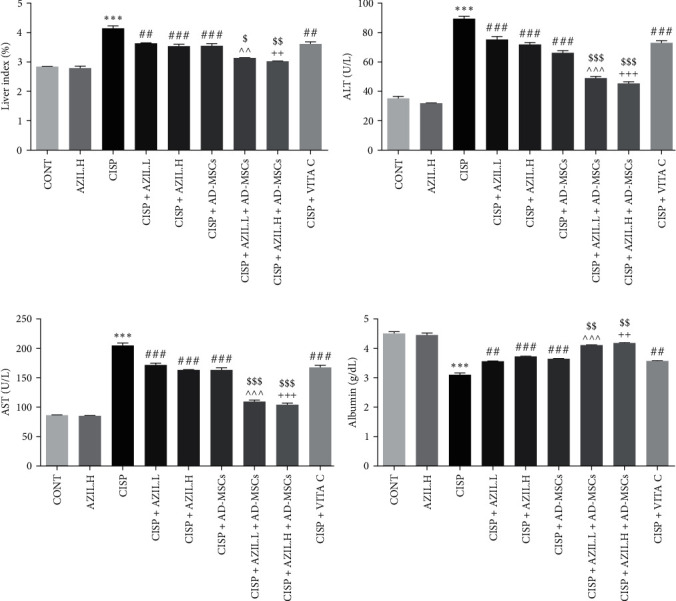
Effects of AZIL and AD-MSCs on hepatic function biomarkers; liver index (a), serum ALT (b), serum AST (c) activities, and serum albumin (d) of CISP-injected rats. Data are represented as mean ± SEM (*n* = 6). Followed by Tukey–Kramer multiple comparisons test, analyses were carried out utilizing one-way ANOVA, where:  ^*∗∗∗*^*p* < 0.001, in comparison with CONT group. ^##^*p* < 0.01 and ^###^*p* < 0.001, in comparison with the CISP group. ^^^^*p* < 0.01 and ^^^^^*p* < 0.001, in comparison with CISP + AZIL.L group. ^++^*p* < 0.01 and ^+++^*p* < 0.001, in comparison with CISP + AZIL.H group. ^$^*p* < 0.05, ^$$^*p* < 0.01, and ^$$$^*p* < 0.001, in comparison with CISP + AD-MSCs group.

Figure 3 shows a marked elevation in liver index and levels of both serum ALT and AST as well as, a significant reduction in serum albumin level were observed following CISP administration as compared to CONT group rats. This hepatocellular toxic impact was nearly altered in all treatment groups (AZIL.L, AZIL.H, AD-MSCs, AZIL.L + AD-MSCs, AZIL.H + AD-MSCs, or VITA C). Interestingly, the coadministration of AZIL.L + AD-MSCs or AZIL.H + AD-MSCs produced the highest change in the aforementioned biomarkers in comparison with the individual administration of AZIL.L, AZIL.H, or AD-MSCs.

### 3.3. Effects of AZIL and AD-MSCs on the Hepatic ROS Level and Oxidative Stress (MDA, GSH, and SOD) Markers

**Figure 4:**
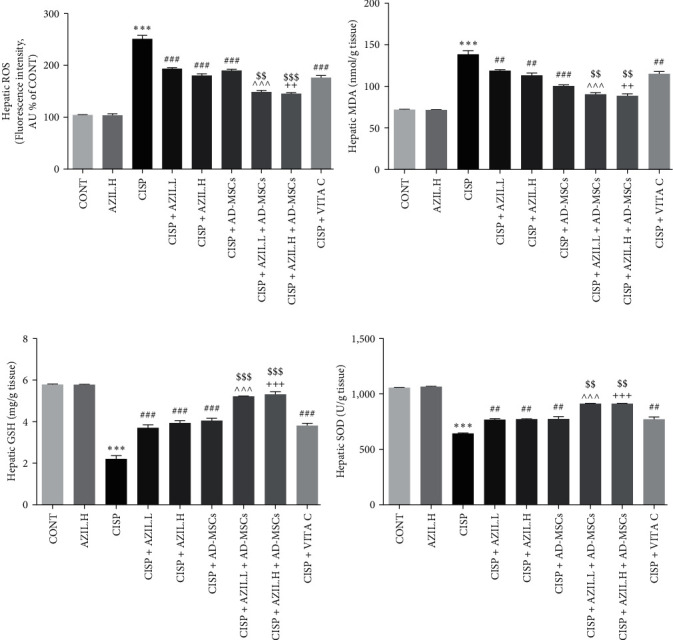
Effects of AZIL and AD-MSCs on hepatic ROS level (a) and oxidative stress indicators of CISP-intoxicated rats; MDA content (b), GSH level (c), and SOD activity (d). Data are represented as mean ± SEM (*n* = 6). Followed by Tukey–Kramer multiple comparisons test, analyses were carried out utilizing one-way ANOVA, where:  ^*∗∗∗*^*p* < 0.001, in comparison with CONT group. ^##^*p* < 0.01 and ^###^*p* < 0.001, in comparison with the CISP group. ^^^^^*p* < 0.001, in comparison with CISP + AZIL.L group. ^++^*p* < 0.01 and ^+++^*p* < 0.001, in comparison with CISP + AZIL.H group. ^$$^*p* < 0.01 and ^$$$^*p* < 0.001, in comparison with CISP + AD-MSCs group.

The injection of CISP led to a substantial rise in the hepatic ROS level (Figure 4(a)) and MDA content (Figure 4(b)), as well as a dramatic reduction in liver GSH (Figure 4(c)) level and SOD (Figure 4(d)) activity as compared to CONT group rats. However, the rats treated with AZIL.L, AZIL.H, AD-MSCs, AZIL.L + AD-MSCs, AZIL.H + AD-MSCs, or VITA C exhibited a marked decline in ROS levels and MDA contents and an elevation in GSH levels and SOD activities in the liver tissue when compared to the CISP-intoxicated rats. The combination treatments have a more significant effect on normalizing ROS levels, MDA contents, GSH levels, and SOD activities. Thus, the effect of the combinations was more notably potent as compared with the effect of the monotherapy treatments.

### 3.4. Effects of AZIL and AD-MSCs on mRNA Expression Levels of the Proinflammatory Cytokines *TNF-α* and *IL-6*

**Figure 5:**
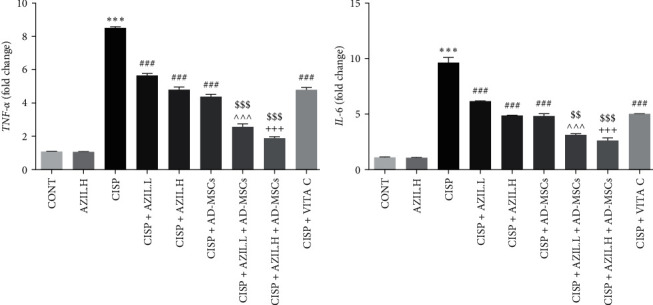
Effects of AZIL and AD-MSCs on the mRNA expression levels of the proinflammatory cytokines; *TNF-α* (a) and *IL-6* (b) in the hepatic tissues of CISP-intoxicated rats. The mRNA expression of different groups was evaluated utilizing qRT-PCR. The expression was displayed relative to the CONT group and normalized to the expression of the related *β-actin* gene. Data are represented as mean ± SEM (*n* = 6). Followed by Tukey–Kramer multiple comparisons test, analyses were carried out utilizing one-way ANOVA, where:  ^*∗∗∗*^*p* < 0.001, in comparison with CONT group. ^###^*p* < 0.001, in comparison with the CISP group. ^^^^^*p* < 0.001, in comparison with CISP + AZIL.L group. ^+++^*p* < 0.001, in comparison with CISP + AZIL.H group. ^$$^*p* < 0.01 and ^$$$^*p* < 0.001, in comparison with CISP + AD-MSCs group.

The inhibitory impact of AZIL or AD-MSCs on the proinflammatory cytokines (*TNF-α* and *IL-6*) in hepatic tissues was evaluated using the qRT-PCR approach. The hepatic mRNA expression levels of *TNF-α* (Figure 5(a)) and *IL-6* (Figure 5(b)) genes were considerably higher in the CISP-intoxicated group than in the CONT group. In the CISP-intoxicated group treated with monotherapy, a considerable down-regulation in the hepatic mRNA levels of examined genes as compared with the CISP-intoxicated group was observed. Furthermore, cotreatment with AZIL.L or H + AD-MSCs resulted in a dramatic down-regulation in hepatic mRNA levels of *TNF-α* and *IL-6* genes over the groups treated with only individual therapy.

### 3.5. Effects of AZIL and AD-MSCs on Hepatic Expression of p-JNK1/2, p-ERK1/2, p-P38, Bax, Bcl-2, and Cleaved Caspase-3 Proteins

**Figure 6:**
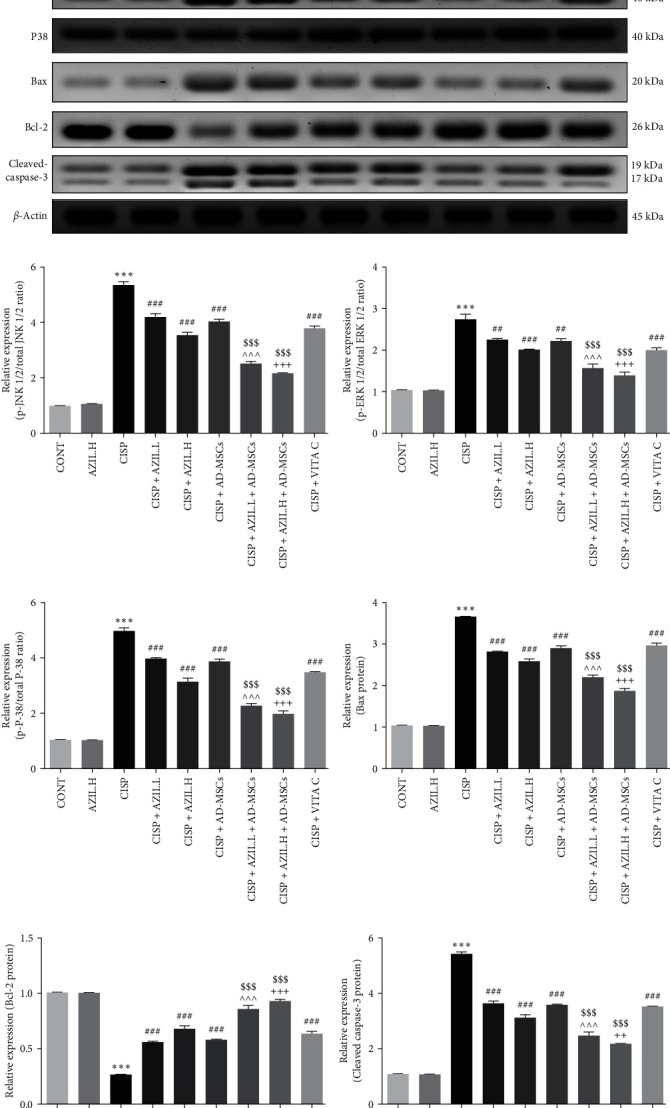
Effects of AZIL and AD-MSCs on hepatic JNK1/2, ERK1/2, p38, Bax, Bcl-2, and cleaved caspase-3 proteins expression of CISP-intoxicated rats. Representative western blot results of the assessed proteins in the liver of the investigated groups (a). Expressions of p-JNK1/2/total JNK1/2, p-ERK1/2/total ERK1/2, p-P38/total P38, Bax, Bcl-2, and cleaved caspase-3, respectively, were displayed densitometrically, utilizing bands in (a) after normalization to the internal CONT *β*-actin, as fold change relative to that of CONT group (b–g). Data are represented as mean ± SEM. Followed by Tukey–Kramer multiple comparisons test, analyses were carried out utilizing one-way ANOVA, where:  ^*∗∗∗*^*p* < 0.001, in comparison with CONT group. ^##^*p* < 0.01 and ^###^*p* < 0.001, in comparison with the CISP group. ^^^^^*p* < 0.001, in comparison with CISP + AZIL.L group. ^++^*p* < 0.01 and ^+++^*p* < 0.001, in comparison with CISP + AZIL.H group. ^$$$^*p* < 0.001, in comparison with CISP + AD-MSCs group.

In comparison to the CONT group, the expression levels of p-JNK1/2/total JNK1/2, p-ERK1/2/total ERK1/2, p-P38/total P38, Bax, and cleaved caspase-3 proteins were dramatically increased in the liver of CISP-intoxicated rats. On the contrary, the expression of Bcl-2 protein in the CISP group was markedly lower than that in the CONT group. The protein expressions of p-JNK1/2/total JNK1/2, p-ERK1/2/total ERK1/2, p-P38/total P38, Bax, and cleaved caspase-3 were remarkably decreased in all treated groups compared to the CISP group. As well, the protein expression of Bcl-2 was noticeably elevated. Intriguingly, the administration of AZIL.L or H + AD-MSCs exhibited considerably significant inhibitory action on the expression levels of p-JNK1/2/total JNK1/2, p-ERK1/2/total ERK1/2, p-P38/total P38, Bax, and cleaved caspase-3 proteins as well as an increasing effect on Bcl-2 protein expression level compared to groups treated with monotherapy (Figure 6).

### 3.6. Effect of AZIL on the Homing Ability of PKH26-Labeled AD-MSCs

**Figure 7:**
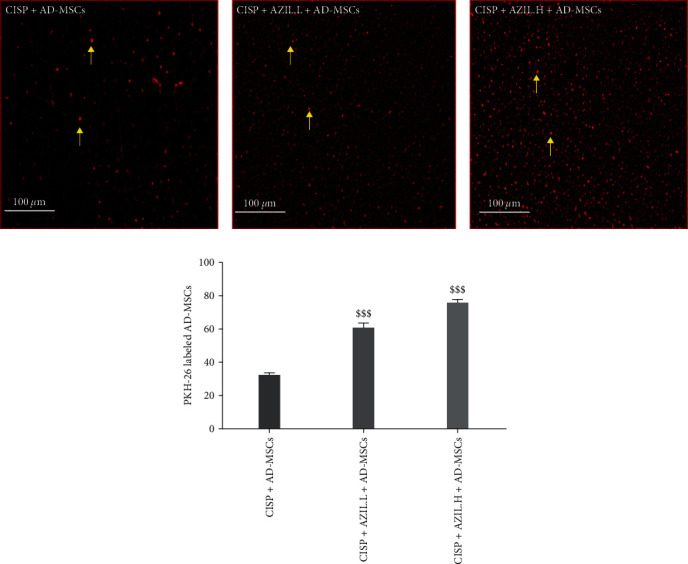
Effect of AZIL on the homing ability of PKH26-labeled AD-MSCs. Immunofluorescent photomicrographs of PKH26-labeled AD-MSCs in the hepatic sections of CISP-injected rats administered with AD-MSCs, AZIL.L, or H + AD-MSCs (a). Quantitative assessment of the average count of PKH26-labeled AD-MSCs (b). The average count of PKH26-labeled AD-MSCs was quantified utilizing Image-J/NIH software. Data are represented as mean ± SEM (*n* = 6). Followed by Tukey–Kramer multiple comparisons test, analyses were carried out utilizing one-way ANOVA, where: ^$$$^*p* < 0.001, in comparison with CISP + AD-MSCs group.

The homing of PKH26-labeled AD-MSCs in the treated groups is represented in Figure 7. Liver tissues of CISP-intoxicated rats treated with AZIL.L + AD-MSCs or AZIL.H + AD-MSCs showed a significant increment of fluorescent MSCs to (60.8 ± 3.3) and (75.5 ± 3.1), respectively, as compared with rats received AD-MSCs only (31.5 ± 2.6). Consequently, the homing ability of AD-MSCs was further enhanced by AZIL.

### 3.7. Effects of AZIL and AD-MSCs on Hepatic Histopathological Changes

**Figure 8:**
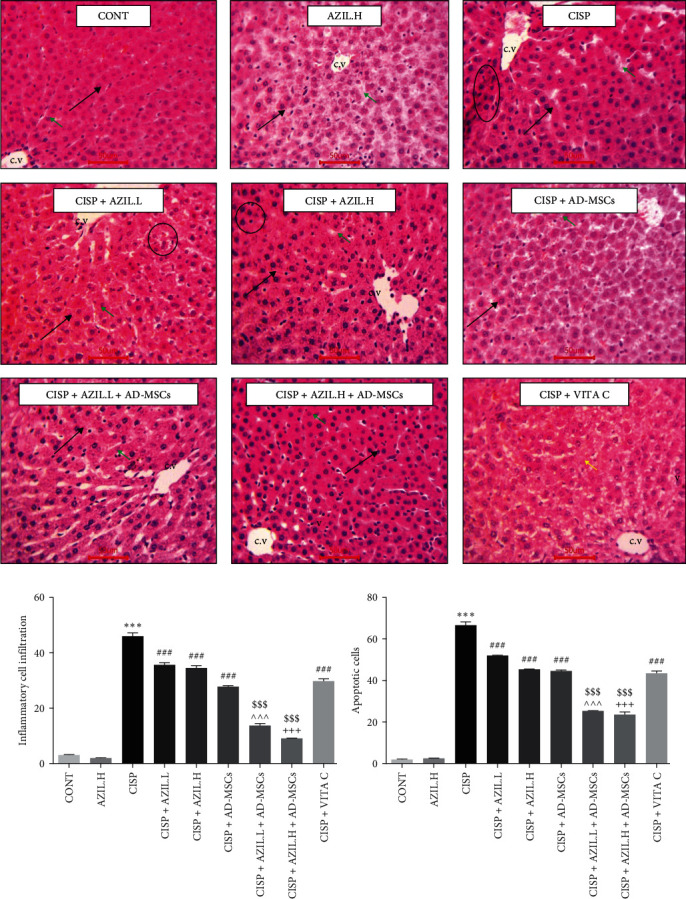
Effects of AZIL and AD-MSCs on hepatic histopathology examinations (a). Representative micrographs of liver sections from CONT as well as AZIL groups showing normal hepatic architecture. The hepatocytes radiated from the central vein (c.v) and separated by blood sinusoids (green arrows). Hepatocytes showing vesicular nuclei (black arrows). CISP group show dilated central vein (c.v) and dilated sinusoids (green arrow). Notice the apoptotic cell (black arrow) and inflammatory cell infiltration (circle). CISP + AZIL.L and CISP + AZIL.H groups showing dilated central vein (c.v) and dilated sinusoids (green arrow). Notice the apoptotic cell (black arrow) and inflammatory cell infiltration (circle). CISP + AD-MSCs group showing dilated blood sinusoids (green arrow) with little apoptotic cell (black arrow). CISP + AZIL.L + AD-MSCs group showing dilated blood sinusoids (green arrow) with little apoptotic cell (black arrow). CISP + AZIL.H + AD-MSCs group showing little apoptotic cells (black arrow) with few vacuolations (v). Liver cells in the CISP + VITA C group appeared with cytoplasmic vacuolations (v). Darkly stained cells (yellow arrow) can be seen (H&E × 400). The average count of inflammatory cell infiltration (b) and the average count of apoptotic cells (c). Data are represented as mean ± SEM (*n* = 6). Followed by the Tukey–Kramer multiple comparisons test, analyses were carried out utilizing one-way ANOVA, where:  ^*∗∗∗*^*p* < 0.001, in comparison with CONT group. ^###^*p* < 0.001, in comparison with the CISP group. ^^^^^*p* < 0.001, in comparison with CISP + AZIL.L group. ^+++^*p* < 0.001, in comparison with CISP + AZIL.H group. ^$$$^*p* < 0.001, in comparison with CISP + AD-MSCs group.

Both the CONT and AZIL.H groups showed normal hepatic architecture. The hepatocytes radiated from the central vein and were segregated by blood sinusoids. Liver cells were seen to have vesicular nuclei. While the CISP group displayed dilated central vein and dilated sinusoids. More numerous apoptotic cells as well as inflammatory cell infiltration were commonly observed in CISP sections. In the CISP + AZIL.L or H groups, dilated central veins, dilated blood sinusoids, apoptotic cells, and inflammatory cell infiltration were noticed. Furthermore, the CISP + AD-MSCs and CISP + AZIL.L + AD-MSCs groups showed dilated blood sinusoids with few apoptotic cells. The CISP + AZIL.H + AD-MSCs group displayed few apoptotic cells with few vacuolations. The liver cells in the CISP + VITA C group exhibited cytoplasmic vacuolations. Notice the darkly stained cells (Figure 8(a)).

### 3.8. Effects of AZIL and AD-MSCs on the Average Count of Inflammatory Cell Infiltration and Apoptotic Cells

In comparison to the CONT group, there was a marked rise in the average count of inflammatory cell infiltration (Figure 8(b)) and apoptotic cells (Figure 8(c)) in CISP-intoxicated rats. By contrast, there was a considerable reduction in the average count of inflammatory cell infiltration and the average count of apoptotic cells in rats treated with AZIL.L, AZIL.H, AD-MSCs, AZIL.L + AD-MSCs, AZIL.H + AD-MSCs, or VITA C as compared to the CISP group. Importantly, the dual administration of AZIL.L + AD-MSCs or AZIL.H + AD-MSCs markedly reduced the above-mentioned parameters when compared to the AZIL.L, AZIL.H, or AD-MSCs groups.

## 4. Discussion

CISP, a commonly administered antineoplastic medication, is beneficial in the treatment of broad types of malignancies. Nevertheless, CISP medical usage is greatly constrained due to its toxicity for various organs [42, 51]. CISP induces cytotoxicity by causing oxidative stress and inflammation, which leads to apoptosis [52, 53]. Many antioxidants and drugs are frequently used in many trials to mitigate CISP-induced liver damage [51, 54]. In addition, AZIL has been demonstrated to have renoprotective properties [25, 55]. Recently, the potential for additional therapeutic properties of already existing [56] or novel [57–59] agents has been thoroughly investigated [60, 61]. This work, for the first time, studied the impact of AZIL and/or AD-MSCs on CISP-evoked hepatotoxicity as well as the proposed molecular basis concerning this effect through exploring the oxidative stress, MAPK, and apoptosis signaling pathways.

In our investigation, CISP injection resulted in a marked elevation in serum ALT and AST activities, which are regarded as the main indicators of hepatocyte damage, a significant increase in liver index, a decrease in serum albumin level, and a remarkable variation in hepatic architecture. In parallel, Man et al. [62] reported that injecting CISP to rats led to a remarkable increase in liver index. This increase might be attributed to increased hepatic weight, which is obviously associated with liver injury [63].

Interestingly, administration of AZIL, AD-MSCs, and their combinations to CISP-treated rats considerably improved all the assessed parameters as well as the liver histological modifications, suggesting less liver damage and better liver function, which may be happened as a result of their antioxidant action and their capacity to limit the release of oxidizing radicals via inhibiting the hepatic endoplasmic reticulum stress, which may be consistent with previous reports [15, 55, 64].

The liver plays a crucial function in removing the ROS generated during normal metabolism through numerous antioxidant system processes [65, 66]. ROS, produced after CISP administration, induce oxidative stress which performs a fundamental role in the development and progression of hepatotoxicity. There are two mechanisms that have been suggested to explain the formation of ROS in CISP-evoked pathological states. First, within a cell, CISP is transformed into a very reactive form that may quickly react with molecules that contain a thiol group, such as GSH which is a well-known cellular antioxidant [67]. Second, CISP may cause mitochondrial disruption and promote ROS formation via the interrupted respiratory chain [68]. The generated ROS allow the oxidative damage of the biological components, including DNA, proteins, and lipids. Thus, the antioxidant defense system is vital to counter oxidative stress and preserving the cell from the potentially harmful consequences of ROS and oxidative impairment [69].

In the current investigation, CISP injection prompted a remarkable rise in the hepatic ROS level and MDA content with concomitant hepatic depletion in GSH concentration and SOD activity, indicating a marked increase in lipid peroxidation (LP) and a concurrent alteration of the equilibrium of oxidants and antioxidants. The reduction of antioxidants and the elevated oxidative stress in the CISP group produced functional impairments in hepatocytes in protein synthesis (albumin particularly) [70], which was decreased in the present study and in agreement with those of El-Hak et al. [63].

In the present study, both doses of AZIL and/or AD-MSCs were able to counteract ROS and reduce LP by lowering the MDA level in parallel with elevating the levels of GSH and SOD, implying a significant improvement of antioxidant defenses. Accordingly, they mitigated hepatic tissue damage by reducing ROS as well as strengthening the antioxidant defensive system inside the liver. This effect may be due to the antioxidant properties of both treatments. According to previous findings, AZIL possesses the aptitude to suppress ROS generation [71, 72]. While MSCs could directly inhibit free radicals by secreting extracellular antioxidant molecules, which might be one strategy for reducing the amount of ROS and promoting hepatic tissue repair [73]. In addition, they showed antioxidant efficacy by preventing LP, raising GSH and SOD levels [74]. Therefore, a combination therapy of AZIL and AD-MSCs was required for optimum antioxidation against CISP-induced hepatotoxicity.

Oxidative stress as well as inflammation are closely correlated, as increased ROS formation triggers an imbalanced immune response and inflammation [75–77]. Furthermore, the massive ROS-inflammatory production may induce permanent cell damage leading to cell death through necrotic and/or apoptotic mechanisms [78, 79]. This clarifies the levels of inflammatory indicators in liver tissues following CISP administration, including the present study's finding that CISP triggered proinflammatory cytokines like *TNF-α* as well as *IL-6*. Similarly, El-Shitany and Eid [42] and Habib et al. [51] concluded that CISP leads to elevated *TNF-α* and *IL-6*, which supports its hepatic inflammatory reaction. In addition, inflammatory cells in liver tissue were clearly noticed in CISP-intoxicated rats, which were evaluated in our work by morphometric analysis. In agreement with El-Hak et al. [63], the presence of inflammatory cells in liver tissue is due to CISP interactions with interstitial liver tissue enzymes and proteins, which interfere with the antioxidant defense machinery and cause the generation of ROS, which may then trigger an inflammatory response. Therefore, the mechanisms underlying the anti-inflammatory properties of AZIL and/or AD-MSCs should be investigated.

The treatment with AZIL, AD-MSCs, or in combination dramatically reduced the expression *TNF-α* and *IL-6* genes, which is thought to be the principal mechanism behind their hepatoprotective impacts against CISP-induced hepatotoxicity. These results were in accordance with our other morphometric measurements, which demonstrated the capacity of AZIL and/or AD-MSCs to diminish inflammatory cells infiltration. A previous study also reported that AZIL down-regulated the expression of *IL-6* and *TNF-α* genes in a renal ischemia/reperfusion injury model in rats [25]. In an oral mucositis experimental model, administration of AZIL dramatically lowered *TNF-α* and IL-1*β* levels while boosting the levels of the anti-inflammatory cytokine IL-10 [80]. Furthermore, AZIL lowered the acute production and release of key proinflammatory cytokines into the circulation as a result of its actions on peripheral macrophages [81]. The inhibitory effect of AD-MSCs on the mRNA expression levels of the proinflammatory cytokines *TNF-α* and *IL-6* in our study was similar to that exhibited in a hepatic ischemia–reperfusion injury in rats [82].

In addition, it is worth noting that MSCs promote liver regeneration by decreasing polymorphic nuclear cell infiltration and vacuolar degeneration in the damaged liver [15]. Besides, they have a strong immunomodulatory effect, which reduces inflammatory responses [83]. As a result, AZIL or AD-MSCs anti-inflammatory properties are crucial in reducing liver inflammation caused by CISP. Of particular importance, the inhibitory effect of the combination therapy of AZIL and AD-MSCs on CISP-induced inflammatory response was more profound relative to the single therapy, which indirectly highlights that their combination may be an effective regimen to achieve the most anti-inflammatory effect.

CISP-produced ROS trigger a variety of downstream proteins that promote apoptosis and necrosis, notably MAPK family members [84]. The MAPK family encompasses the following serine/threonine kinase proteins, JNK, ERK, and p38 that are implicated in cell development and differentiation and have been connected to inflammation, apoptosis, and cell death [85]. CISP turns the balance of pro- and anti-apoptotic proteins in favor of the proapoptotic pathway [86]. It activates Bax while diminishing Bcl-2 protein and subsequently leads to the liberation of cytochrome c into the cytoplasm, where it triggers caspases and results in apoptotic cell death. Consistently in this work, phosphorylation of JNK 1/2, ERK1/2, and p38 in hepatic tissues was demonstrated in CISP-intoxicated rats. These results are consistent with those of Omar et al. [53] who reported that administration of CISP increased the phosphorylation of all three proteins of the MAPK family. Collectively, these findings suggest that the MAPK signaling pathway is involved in the induction of hepatotoxicity by CISP. Hence, the MAPK pathway could act as a potential target to probe new therapeutic strategies to mitigate CISP-induced hepatotoxicity.

On the other hand, these raised phosphorylation levels were diminished by treatment with AZIL and/or AD-MSCs. These findings supported previous studies that demonstrated that AZIL treatment suppressed the activation and phosphorylation of ERK1/2, JNK1/2, and p38 proteins in MAPK-mediated osteoclastogenesis in vitro [87] and in a renal ischemia/reperfusion injury model in rats [25]. Thereby, the hepatoprotective effect of AZIL against CISP-induced hepatotoxicity is exerted through regulation of the MAPK signaling pathway. In addition, Huang et al. [88] reported that AD-MSCs suppressed phosphorylation of the ERK and JNK proteins in acetaminophen-induced acute liver failure in vivo; these were in line with the current work. In the current study, elevated expression of Bax and cleaved caspase-3 proteins and lowered Bcl-2 protein expression in the hepatic tissues of CISP-intoxicated rats in parallel with previous studies [89, 90], together with a rise in the average count of apoptotic cells in the morphometric analysis, suggested significant apoptosis in these rats. Our findings revealed that AZIL and/or AD-MSCs, by attenuating the MAPK pathway, diminished the average count of apoptotic cells and the expression of cleaved caspase-3 and Bax proteins, as well as raised Bcl-2 protein expression, leading to antiapoptotic action in the liver tissues of CISP-intoxicated rats treated with them. These findings corroborate those of Garg et al. [91] in which AZIL increased Bcl-2 protein expression and decreased the expression of Bax and caspase-3 proteins in an in vivo model of myocardial ischemia–reperfusion injury and exerted an antiapoptotic effect. In addition, a recent study showed that AD-MSCs have the potential to reduce apoptosis in liver cells following acute liver injury induced by diclofenac sodium in rats by increasing the expression of the anti-apoptotic gene *Bcl-2* and decreasing the expression of the proapoptotic gene *Bax*, as well as the cleaved caspase-3 protein, which is a potential executioner of apoptosis [15].

These promising findings were in accordance with those that demonstrated that angiotensin receptor blockers control the oxidative stress and apoptosis that are produced by angiotensin II in mesangial cells [92]. In addition, MSCs could have a direct therapeutic effect by replacing damaged cells or an indirect therapeutic effect by triggering cellular regeneration, as well as stimulate the production of factors with antiapoptotic, anti-inflammatory, mitogenic, or angiogenic activities [93, 94].

Finally, it was found that AZIL at a low dose showed no significant difference in biochemical, molecular levels, and histological examination compared to those at a high dose on CISP-induced hepatotoxicity. So we recommend using the low dose in order to reduce cost-effectiveness. In addition, there is no significant difference between AZIL.L + AD-MSCs and AZIL.H + AD-MSCs groups in all items assessed in the present study except cell homing of AD-MSCs. But they showed similar significant changes when compared to AD-MSCs treatment alone.

More interestingly, in the present work, the combination of AZIL and AD-MSCs improved all the previously studied parameters more effectively than AZIL or AD-MSCs alone. These findings revealed the enhancement of the therapeutic capability of AD-MSCs by AZIL via increasing cell homing of AD-MSCs to the injured liver, which was observed in this study by fluorescence microscope. Effective homing of MSCs into injured tissue has been recognized as one of the most critical obstacles to successful stem therapy as demonstrated by Habib et al. [19] who revealed that exenatide promoted the homing capability of AD-MSCs in the kidney tissues of diabetic rats. In addition, Abdelhafez et al. [95] demonstrated that VITA C and *N*-acetylcysteine enhanced the homing capability of bone marrow-derived MSCs in the pancreatic tissue.

The current study is the first one that demonstrated the new hepatoprotective effect of AZIL, explaining, on molecular levels, the mechanism underlying this new effect, which could represent a novel therapeutic strategy for attenuating CISP-induced hepatotoxicity. Moreover, these findings highlight the significance of AZIL in increasing the therapeutic efficacy of AD-MSCs in reducing CISP-induced hepatotoxicity by attenuating oxidative stress, inflammation, MAPKs, and apoptotic pathways, lighting the way for enhanced cell-based therapies in general and chemotherapeutics in particular.

## 5. Conclusions

Our findings demonstrated for the first time that dual administration of AZIL and AD-MSCs may provide markedly greater hepatoprotection against CISP-induced hepatotoxicity than AZIL or AD-MSCs usage alone. They were effective in reducing CISP-evoked hepatotoxicity by suppressing oxidative stress, inflammation, MAPKs, and apoptotic pathways. AZIL considerably improved the hepatoprotective capability of AD-MSCs against CISP-evoked hepatotoxicity, recommending this strategy to increase the therapeutic potential of AD-MSCs. However, additional clinical research is required to validate such preclinical outcomes.

## Data Availability

All the data used to support the findings of this study are included within the article.

## Abbreviations

AD-MSCs: Adipose tissue-derived mesenchymal stem cells
ALT: Alanine aminotransferase
AST: Aspartate aminotransferase
AZIL.L or .H: Azilsartan low or high
Bax: Bcl-2-associated X protein
Bcl-2: B-cell lymphoma 2
CISP: Cisplatin
CONT: Control
ERKs: Extracellular signal-regulated kinases
GSH: Reduced glutathione
H&E: Hematoxylin and Eosin
*i.p.*: Intraperitoneal
IL: Interleukin
JNK: c-Jun N-terminal kinase
LP: Lipid peroxidation
MAPKs: Mitogen-activated protein kinases
MDA: Malondialdehyde
MSCs: Mesenchymal stem cells
ROS: Reactive oxygen species
SOD: Superoxide dismutase
TNF-*α*: Tumor necrosis factor-alpha.
